# Genome sequences derived from pig and dried blood pig feed samples provide important insights into the transmission of African swine fever virus in China in 2018

**DOI:** 10.1080/22221751.2019.1565915

**Published:** 2019-02-27

**Authors:** Xuexia Wen, Xijun He, Xiang Zhang, Xianfeng Zhang, Liling Liu, Yuntao Guan, Ying Zhang, Zhigao Bu

**Affiliations:** a State Key Laboratory of Veterinary Biotechnology, Harbin Veterinary Research Institute, Chinese Academy of Agricultural Sciences, Harbin, People’s Republic of China; b College of Animal Science and Veterinary Medicine, Shenyang Agricultural University, Shenyang, People’s Republic of China

**Keywords:** African swine fever virus, genome sequence, China

African swine fever (ASF) is a devastating viral haemorrhagic disease of domestic pigs and wild boars. African swine fever virus (ASFV) is a double-stranded DNA virus and is the only member of the family *Asfarvidae*, genus *Asfivirus.* ASF was initially reported in Kenya in 1921 [1]. In the 1950s, ASFV rapidly spread throughout Europe and was eradicated in many countries, with the exception of Sardinia, by the mid-1990s [2,3]. In 2007, an ASF case was reported in the country of Georgia (we refer to the virus as GA/2007 throughout this report); since then, ASFV has become prevalent in Russia and spread to other European countries [4–7].

The length of the genome of ASFVs varies depending on the isolate, but ranges from 170 to 193 Kb; 150 to 167 open reading frames (ORFs) have been detected from various strains [8–11]. The ASFVs have been divided into 24 different genotypes based on their *B646L* gene, which encodes the capsid protein p72 [12]. The GA/2007 strain and the subsequent isolates in European countries all belong to genotype II, although additional genetic variations have occurred during its decade-long circulation in Europe. An insertion of a 10-nucleotide “TATATAGGAA” tandem-repeat sequence (TRS), located between *I73R* and *I329L*, was first found in wild boar ASFVs in Lithuania and Poland in 2014 [13], and this insertion is now recognized as a new sub-genotype marker [12]. In 2017, serial ASF outbreaks occurred in Siberia near to the Russia-China border [4], and on 3 August 2018, the first ASF case in China was reported on a pig farm in the suburb of Shenyang, Liaoning province [14]. Zhou et al. and Ge et al. detected the viral genome of the ASFV in the pigs of the first Shenyang ASF outbreak, and Miao et al. recently uploaded the full genome sequence ASFV-SY18 (GenBank: MH766894.1) of the ASFV that was responsible for the first ASF outbreak in China.

On 5 September 2018, ASF outbreak was reported in a farm in the city of Jiamusi, Heilongjiang province [14]. There were 87 pigs in the farm, amongst which 39 pigs were sick and 12 of them died. Spleen samples from three dead pigs were collected for laboratory tests. All the three samples were found ASF positive as confirmed by Q-PCR. We extracted the viral DNA from the spleen sample of one dead pig and sequenced the full genome.

Pig blood is commonly collected from pig slaughterhouse and then used as feed for pigs and other animals as an important protein source. To investigate if the dried blood pig feeds were contaminated by ASFV, 20 batches of dried blood produced by a company in Heishan county, Liaoning province and 1 batch of dried blood collected in Heilongjiang produced by a company in Hebei province were sampled and tested for ASFV genome by Q-PCR, and all of them were found ASFV positive. We selected one batch of the dried blood sample from Liaoning and sequenced the full genome of ASFV.

The full genome of ASFVs was sequenced using a segmentation PCR strategy where 86 segments (each about 2400 bp in length, covering the entire genome) were amplified by using a set of primers that were designed on the basis of the genome sequence of GA/2007 (GenBank: FR682468.1). Adjacent segments shared an overlap of 100–200 bp to eliminate sequence generated by the primers. The 5′ and 3′ terminal sequences were determined by Genome Walking [15]. The purified DNA fragments were sequenced on an Applied Biosystems DNA analyzer ABI 3500, and the nucleotide sequences were edited using the Seqman module of the DNAStar package.

The expected DNA fragments were successfully amplified and the sequence results revealed that the disease pig sample and the dried blood sample carried ASFVs with identical genome sequences. We named these two viruses Pig/HLJ/2018 and DB/LN/2018, respectively (Genbank: MK333180 and MK333181). To investigate the origins of the viruses in our samples, we performed a detailed comparison of our sequences with the sequences of ASFV-SY18, GA/2007, and other ASFVs reported in European countries in recent years. We found that our sequences showed the highest similarity with the sequence of an ASFV isolated in Poland (GenBank: MG939588.1), and we refer to this Polish virus as PoL/2017 in this report.

**Figure 1.**
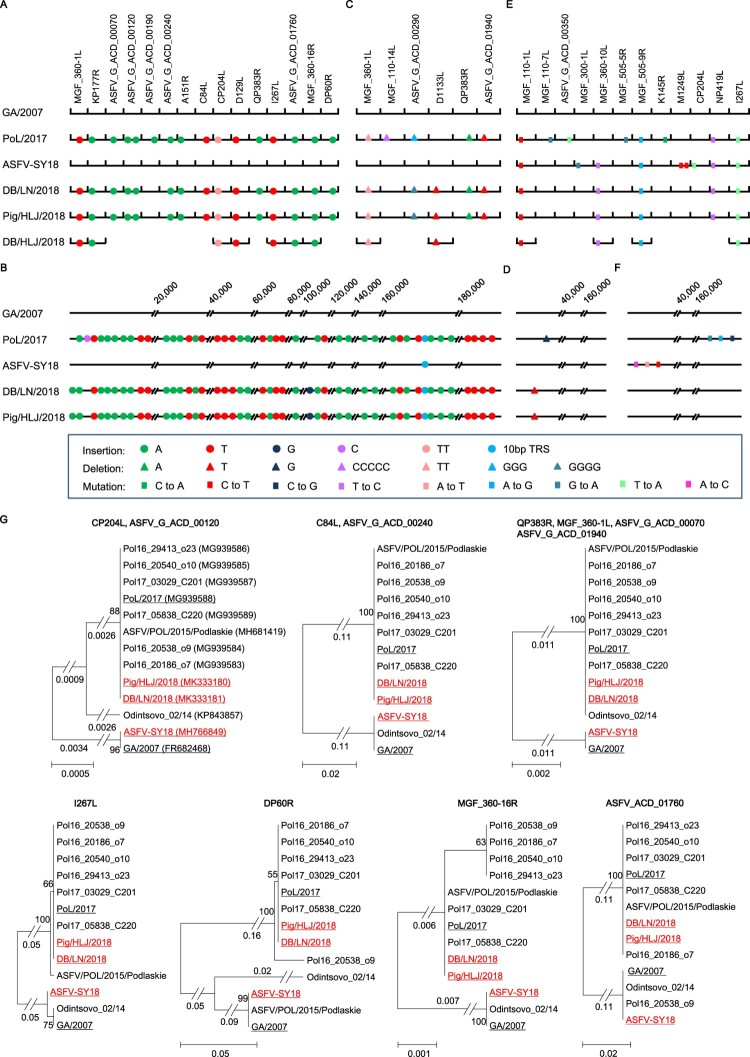
Nucleotide insertions, deletions, and mutations of different Africa swine fever viruses (ASFVs) detected in China. The genome sequences of three Chinese ASFVs and the Polish virus PoL/2017 were compared with the Georgia 2007 isolate GA/2007 virus. (A) Nucleotide insertions within the open reading frames (ORFs). (B) Nucleotide insertions in the noncoding region. (C) Nucleotide deletions within the ORFs. (D) Nucleotide deletions in the noncoding region. (E) Nucleotide mutations within the ORFs. (F) Nucleotide mutations in the noncoding region. The names of the ORFs are shown on the top of each panel. The numbers on the top of the panels indicate the relative genome positions. Eleven ORFs of the DB/HLJ/2018 were sequenced, and the insertions, deletions and mutations in these ORFs are shown in panels A, C, and E, respectively. (G) Phylogenetic trees of proteins encoded by each ORFs. Only one tree is shown when several ORFs have similar trees, but all of the names of the ORFs are shown on the top of the tree. The access numbers of the sequences are shown in the brackets of the first tree. The five viruses shown in panels A–F are underlined in the trees.

The lengths of the genome sequences of Pig/HLJ/2018 and DB/LN/2018 are both 189,404 bp, and the length of PoL/2017 is 189,401 bp. The GA/2007 sequence is 189,344 bp long, excluding the terminal inverted repeats and cross links [9]. The ASFV-SY18 sequence is 189,354 bp long (GenBank: MH766894.1), which is very similar to the sequence of GA/2007, with only 12 nucleotide differences detected in the entire genome, excluding the 10-nucleotide TRS insertion (Figure 1). Compared with the sequences of GA/2007 and ASFV-SY18, nucleotide insertions, deletions, and mutations had occurred in multiple positions in the genomes of the PoL/2017, Pig/HLJ/2018, and DB/LN/2018 viruses.

Compared with the GA/2007 virus, the Pig/HLJ/2018 and DB/LN/2018 viruses have 16 nucleotides inserted at 15 positions located in 14 different ORFs (Figure 1(A)). These insertions include 10 single As, four single Ts, and one double T (Figure 1(A)), which were also detected in the PoL/2017 virus, but none appeared in the ASFV-SY18 virus (Figure 1(A)). Pig/HLJ/2018 and DB/LN/2018 have 54 nucleotides inserted at 45 positions in noncoding region, and these insertions include 25 single As, 18 single Ts, 1 single G, and the 10-bp TRS (Figure 1(B)). Of note, most of the insertions (24 of the 25 single As, the 18 single Ts, and the 10-bp TRS) also appeared in the PoL/2017 virus, but only the 10-bp TRS was reported in the ASFV-SY18 virus (Figure 1(B)).

Compared with the GA/2007 virus, Pig/HLJ/2018 and DB/LN/2018 have nine nucleotides deleted at five positions of five different ORFs (Figure 1(C)) and a single T deletion in the noncoding region (Figure 1(D)). The deletions in the five ORFs include a double T, a quadruple G, two single Ts, and a single A (Figure 1(C)). Three of the five deletions, including the double T and the two single Ts in the ORFs also appeared in the PoL/2017 virus (Figure 1(C)). The PoL/2017 virus has a five C deletion in the ORF of MGF_110_14L, whereas our viruses do not have this deletion. The PoL/2017 virus has a three G deletion in the ORF of ASFV_G_ACD_00290, whereas the Pig/HLJ/2018 and DB/LN/2018 viruses have a quadruple G deletion at the same position (Figure 1(C)). We were surprised that ASFV-SY18 does not contain any of the insertions or deletions observed in our viruses, except for the 10-bp TRS (Figure 1(C,D)). One possible explanation for this is that different ASFVs may have been introduced into the pig population in China; another explanation may be that the genome of ASFV-SY18 was not correctly compiled due to the sequencing method used.

In addition to insertions and deletions, we found five nucleotide mutations in five different ORFs in Pig/HLJ/2018 and DB/LN/2018 (Figure 1(E)). The ASFV-SY18 virus has all five of these mutations and four other mutations in three other ORFs (Figure 1(E)). The PoL/2017 virus has four of the five mutations detected in our viruses and four other mutations in four other ORFs that differ from the ones reported in the ASFV-SY18 virus (Figure 1(E)). Compared with the GA/2007 virus, we did not find any nucleotide mutations in the noncoding regions of the two viruses we sequenced, but the ASFV-SY18 and PoL/2017 each has three mutations in different noncoding regions (Figure 1(F)).

The nucleotide insertions, deletions, and mutations in the genomes resulted in changes in 17 ORFs in the Pig/HLJ/2018 and DB/LN/2018 viruses compared with the GA/2007 virus: one ORF reported for GA/2007 does not exist in Pig/HLJ/2018 or DB/LN/2018; in addition to amino acid mutations, eight ORFs had deletions, ranging from 7 to 90 AAs in length, and seven ORFs had insertions, ranging from 5 to 150 AAs in length (Supporting Table S1). We then performed a phylogenetic analysis of the ORFs of the 3 viruses isolated in China together with the ORFs of 10 other genotype II viruses available in the public database, and found that 12 ORFs of these viruses formed distinct branches: GA/2007 and ASFV-SY18 are in one branch, and the Pig/HLJ/2018 and DB/LN/2018 viruses are in a separate branch of the phylogenetic trees. Of note, most of the viruses isolated between 2014 and 2017 in European countries are also in the group with the Pig/HLJ/2018 and DB/LN/2018 viruses (Figure 1(G)).

The genotype II ASFVs have continuously evolved since their detection in Georgia in 2007. Our study demonstrated that nucleotide insertions and deletions have frequently occurred in ASFVs and some genome changes have resulted in alteration of ORFs. Our phylogenetic analysis indicated that alterations in some ORFs appeared in ASFVs in Europe as early as 2014. Most of the ORF changes in our viruses were also detected in Polish viruses isolated between 2015 and 2017. It remains to be investigated how these changed ORFs affect the properties of ASFV.

In summary, we analysed the genomes of ASFVs derived from a pig sample and a dried blood pig feed sample in China and found that the two viruses are identical. Partial sequence analysis confirmed that dried blood pig feed sample collected in Jiamusi, Heilongjiang was also contaminated with a similar virus; we named this virus DB/HLJ/2018 (GenBank: MK333182 to MK333192) (Figure 1(A)). A live virus was isolated from the pig sample and was proved to be highly lethal and transmissible in pigs (unpublished data). Although we were not able to isolate live virus from the dried blood pig feed samples we tested, this does not mean that ASFV is inactivated in all of the dried blood pig feed currently used in China, which is highly likely a source for the current ASFV spread. Therefore, it is important to inspect pig feed to prevent further ASF outbreaks.
